# Supplementary material for: Are carbon emissions trading and green financial instruments synergistic? -Comprehensive quantitative research based on content analysis
Source: PLoS One. 2024 Mar 7;19(3):e0298601. doi: 10.1371/journal.pone.0298601 (PMC10919601; doi:10.1371/journal.pone.0298601)
Supplement: S1 Data — (ZIP) [file pone.0298601.s001.zip › Data/地级市Z_F工作报告.zip!/║┌┴·╜¡/║╫╕┌╩╨/└┤╫╘╣1⁄2╓┌║┼í╛┬φ┐╦╩2╛▌═°í┐_2015.docx]

各位代表：

　　现在，我代表市人民政府，向大会报告工作，请予审议。并请各位政协委员和其它列席人员提出意见。

　　一、2014年工作回顾

　　过去的一年，是我市发展进程中极其艰难的一年。面对严峻复杂的经济形势、集中显现的社会矛盾和接踵而至的困难问题，在省委、省政府和市委的坚强领导下，我们主动适应经济发展新常态，沉着应对困难和挑战，妥善处理矛盾和问题，在困境中砥砺前行，在逆势中谋求发展。经过各级党政组织和广大干部群众的共同努力，实现了民生持续改善、社会安定和谐、各项事业稳步发展。地区生产总值实现259亿元，下降9.7%；固定资产投资86亿元，下降43.9%；规模以上工业增加值38.3亿元，下降39.5%；公共财政收入16.8亿元，下降18%；社会消费品零售总额108.5亿元，增长1.5%；外贸进出口总额1.39亿美元，增长70.7%；城镇居民人均可支配收入18116元，增长2.8%；农村居民人均可支配收入11463元，增长10.3%。虽然经济总体处于低位运行，但主要经济指标降幅收窄，地区生产总值、规模以上工业增加值和公共财政收入降幅比上半年分别收窄13、12和21个百分点，我市经济发展基本面向好。特别是在主体税源大幅下降、刚性支出不断增加的情况下，通过采取一系列的增收节支措施，实现了保工资、保运转、保民生、保重点。教育、卫生、社保等民生支出占财政总支出的比重上升了1.8个百分点，按照机关事业人员津贴补贴标准上限提高了工资水平，城市低保月标准提高到430元。

　　——过去的一年，面对城市转型的紧迫需求，我们坚持抓招商、上项目，推动了经济发展后劲增强。坚持长远谋事，把产业项目建设作为解困发展、转型升级的重要引擎，对“十大重点产业”逐一分析市场潜力、投资机会、合作项目和空间布局，形成了产业投资发展潜力报告，充实完善了项目库，全力招商，大上项目，统筹推进了一批打基础、利长远的重大项目，为我市长远发展奠定了基础。在全省率先启动了新一轮产业项目建设年活动，开展了项目“四比”竞赛和两次集中开工活动，全年共开复工项目157个，产业项目投资占固定资产投资比重达到52.7%，新上项目非煤比重超过85%。大力发展煤电化工产业，煤焦化及副产品加工利用、煤制化肥、煤基多联产、煤制气、煤转电等产业链条初步形成，中海油煤制化肥项目将在今年3月份正式生产，征楠煤化工内部形成全产业链，鹤翔新能源、中石化煤基多联产、2x300MW热电联产等项目加快推进。快速推进石墨深加工产业，石墨电池负极材料、密封材料、阻燃材料、锂离子蓄电池、胶体石墨等产业链条加快构建，黑鑫石墨纸项目竣工，中铁资源集团与深圳贝特瑞合作项目正式签约，中植集团与贝特尔、检顺公司与杉杉科技合作项目正在推进。迅速壮大绿色食品产业，稻米、玉米、大豆深加工和畜牧养殖、林产品加工等产业链条继续延伸，经纬公司阿拉伯糖项目竣工，万源精炼米糠油、宝泉白羽肉鸡深加工等项目加快建设，华润五丰项目正式签约。8种农产品入选“全省第二届消费者最喜爱的百种绿特色食品”。加快培育科技新兴产业，形成了一批科技含量和附加值高、有竞争力、有市场潜力的产业项目，具有国家专利的鹏程科技吸音板项目建成投产，全国领先的秀泉科技氧化法制浆项目第一条生产线即将竣工，奥太新能源、吉阳光伏等项目扎实推进。招商引资成果丰硕，进一步动员各县区、各部门和社会各界力量，把引进资金、技术和人才作为核心内容，把央企、民企和跨国公司作为主攻方向，通过“香港招商会”、“央企对接会”、“民企龙江行”、“中俄博览会”和赴“京津唐”、“珠三角”、“长江经济带”招商等活动，招引了一批投资额度大、产业带动强、税收贡献多的项目。全年共引进项目187个，总投资289.7亿元，到位资金80.5亿元。

　　——过去的一年，面对经济下行的严峻形势，我们坚持抓运行、遏下滑，推动了整体经济逐步趋稳。坚持科学干事，把加快发展作为解决鹤岗一切矛盾、困难和问题的根本出路，认真分析研判，采取切实措施，全力以赴做精农业、做强工业、做活三产，进一步激活存量，做大增量，促进三次产业协调发展。三农工作稳步推进。市内粮食产量突破30亿斤，创历史最高，水稻种植比重继续保持全省市地之首。农民专业合作社达到347家。建设了三条百里现代农业示范带，辐射带动21个示范区。新建水稻智能催芽车间17个、育秧小区44个和育秧大棚1638栋。新建温室大棚200栋、果蔬储藏窖1.1万平方米，菜篮子工程得到进一步保障。畜牧业发展良好，“两牛一猪”存栏分别增长7.8%、3.6%和8.4%。投资5.38亿元，推进农田水利建设工程22项，关门嘴子水库被确定为全国重大水利工程。向上争取3.2亿元，实施了6个土地整治项目。转移农村劳动力6.2万人（次），实现劳务收入7.6亿元。农村土地承包经营权确权试点工作有序推进。萝北县被认定为国家现代农业示范区。绥滨县中兴村被评为“2014年全国百个最美休闲乡村”。工业经济困中企稳。加大扶企助企力度，实行市级领导包企责任制，成立了企业投诉中心，落实了中小微企业发展支持政策，完善了企业助保金贷款风险补偿机制，累计为中小企业投放贷款5.8亿元，亏损企业由年初的73户减至37户，新增规模以上企业15户。第三产业繁荣活跃。大力发展商贸流通业，顺翔、德翔、陆港等物流项目和益华城市综合体项目有序推进。努力壮大旅游业，开发了精品旅游线路，萝北雪乡生态园项目正式启动，龙江三峡被列入省文化旅游产业重点项目，鹤岗国家森林公园、清源湖景区升级为国家4A级景区，黑龙江流域博物馆被命名为省级爱国主义教育基地，金顶山风景区被评为省级地质公园。积极发展金融业，金融机构各项存贷款余额487亿元和353亿元,同比增长3.2%和14.5%，鹤翔新能源、三江平原米业、正丰小额贷款公司在上海股权交易中心Q板挂牌融资。不断扩大对外经贸合作，与66个国家和地区发生贸易往来。与俄方正式签署了经济合作区共建协议，启动了入境落地签证和出境异地办照业务，口岸过货量比上年翻了两番。

　　——过去的一年，面对财政严重短收、刚性支出增加的现实压力，我们坚持抓重点、保底线，推动了民生事业持续改善。坚持务实做事，把保障和改善民生作为一切工作的出发点和落脚点，不断加大民生投入，尽心竭力、全心全意地为群众办实事、做好事、解难题，真正让改革发展的成果惠及于民。民生工程深入实施。积极推进“三个千人计划”，城镇新增就业2.28万人，城镇登记失业率控制在4%以内。为有创业愿望人员发放小额担保贷款、农村妇女创业贷款6871万元，累计支持1487人创业再就业。实施“五险”扩面，新增参保人员2.4万人，企业退休人员养老金达到1592元。城镇居民、职工医疗费年度最高支付限额分别提高到8万元、20万元，大病统筹报销标准分别提高到75%和87%，城镇基本医疗保险住院医疗费实现本地、部分地区异地即时结算。向2.2万停产煤矿职工发放救助金4466万元，特别是在元旦、春节期间，筹资2亿元，救助群众16.1万人。全市公办和民办养老机构、日间服务中心达到191个，每千名老人拥有养老床位30张。城市社区建设达标率达到70%。坚持“以安置促建设，以建设保安置”，煤矿棚改住宅楼竣工7012套，回迁安置3181户，城市棚户区改造2238户，公共租赁房开复工7246户，农村泥草（危）房改造1950户，因洪灾倒塌和严重损毁的2513户房屋修复重建全部完工。社会事业长足进步。医疗服务日趋完善，市中医院分院、市精神病院、120急救中心、医疗卫生用品洗涤中心等投入使用，市妇幼保健院新址大楼主体完工。人均基本公共卫生服务经费标准提高到35元，新农合人均筹资标准达到390元。县级公立医院改革进一步深化，乡镇卫生院规范化建设率达到90%。实施卫生惠民工程，累计为贫困群众减免医药费528万元。落实基本药物制度，让利群众611万元。全面落实计划生育政策，兑现了城镇无业人员独生子女父母奖励费。教育发展日趋均衡，标准化学校达到61所，学前三年教育入园率达到80%，特教中心被认定为国家级残疾人职业培训基地，鹤岗师专“专升本”考取率连续6年高于全省平均水平。文体生活日趋丰富，农家书屋实现全覆盖，纪实情景剧《时代楷模孙波》在全省新剧目展演中获得8个奖项，开展了“欢乐鹤岗行”等文化下基层活动，实现了各类文化、体育场馆免费开放。成功举办“四国女篮争霸赛”，积极参加省第十三届运动会，累计获得奖牌189块。社会形势持续稳定。严格落实安全生产“四个责任”，开展了安全生产大检查、大排查、大整治，全力推进煤矿整顿关闭工作。创新实施“六个为主”信访处置原则，信访案件办结率接近90%。不断加强社会治安综合治理，先后开展“打四黑、除四害”等10余个专项行动，刑事案件立案率下降12%，命案综合破案率达到120%。应急体系建设全面加强，应对突发事件的能力不断提升。

　　——过去的一年，面对基础设施差、历史欠账多的实际状况，我们坚持抓建设、强管理，推动了城市功能不断提升。坚持竭力办事，按照科学规划、建管并重的原则，从解决人民群众最关心、最期盼、最受益的事情抓起，狠抓基础设施配套和城市功能完善，行路难、吃水难、供暖难和环卫难等问题得到有效解决，城市建设管理水平进一步提升。城市规划不断完善。启动了城市总规修改，编制了采煤沉陷区土地综合治理、新型城镇化、棚户区改造等6个专项规划，编制控详和修详规划107项。基础设施建设不断加强。鹤佳铁路扩能改造项目已纳入国家铁路总公司部省会商纪要。鹤岗机场已经纳入全省民用支线机场布局规划。投资1.2亿元，建设东山路等8条主次干道，建设改造育才路、工农路等112条街巷道路，升级改造公园广场7个。启动了市政供水系统净水厂升级改造工程，与中国水务集团合作成立了鹤岗三立水务有限公司。加强热源建设，热力公司四期和万隆热电联产项目开工建设，改造老旧管网20公里。总投资1.67亿元的城市生活垃圾处理场主体工程完工。大力整顿建筑、房地产开发市场秩序，严肃查处各类违法违规行为，将27个工程项目和企业列入黑名单。建立了“弃管楼”管理长效机制，整治“弃管楼”1344栋。水生态文明城市建设试点全面启动，淘汰黄标车及老旧车辆2307台、小锅炉19台，新建4个PM2.5空气自动监测站，空气质量达到国家二级标准以上天数327天。投资2500万元的鹤岗国家气象观测站及市气象灾害预警中心建成投入使用。全市森林覆盖率达到45.3%，太平沟自然保护区晋升为国家级自然保护区。新建农村饮水安全工程28处。全面加快美丽乡村建设，成功申报25个省级生态村，省三星级以上美丽乡村达到66个。

　　——过去的一年，面对政务服务、依法行政、作风建设等方面存在的实际问题，我们坚持抓提升、促转变，推动了“三型”政府建设。坚持依法依规行事，切实把加强政府自身建设摆在突出位置，适应政府管理创新要求，加快职能转变，不断改进工作手段、工作方法和工作作风，努力提升政府工作的效率、能力和水平。围绕打造服务政府，不断提升政府行政效能。全市精简了196项行政审批事项，占原审批事项的53%。进一步整合工作流程，压缩审批时限，完善市人民办事中心功能，开辟了重大产业项目审批“绿色通道”，实行联合审批、“保姆式”服务。改进市长专线办理流程，办结率、市民满意率大幅提升。着眼于转变政府职能、理顺职责关系，稳步推进政府机构改革和事业单位分类改革，政府工作部门由35个整合为32个。围绕打造法治政府，不断提升依法行政水平。认真执行市人大及其常委会的决议，自觉接受市人大的法律监督和工作监督，主动接受市政协的民主监督，虚心听取各民主党派、工商联、无党派人士和各人民团体的意见建议，全年共办理人大代表建议91件、政协提案63件，建议提案办理工作实现由答复型向落实型转变。围绕打造廉洁政府，不断提升廉洁从政能力。深入开展了党的群众路线教育实践活动，切实查纠“四风”问题，清理了违规公务用车、超标办公用房和“吃空饷”人员。严格贯彻中央和省、市委“八九十项”规定，“三公”经费支出同比明显下降。加快推进权力运行公开化、规范化，政务公开、司法公开和各领域办事公开制度进一步完善。加大了审计监督、行政监察力度，严格落实廉政建设责任制，强化责任追究，推动了廉政建设进一步加强。

　　市场监管、统计、物价、外侨、老龄、双拥、地震、司法行政、档案、民兵、预备役等工作取得新进展，民族宗教、妇女儿童、残疾人、红十字等事业取得新进步。

　　各位代表，过去的一年，在如此严峻的形势下，我们取得这样的成绩实属来之不易。这是省委、省政府和市委正确领导的结果，是市人大、市政协监督支持的结果，是全市上下迎难而上、砥砺前行、拼搏实干的结果。在此，我代表市人民政府，向全市人民，向各民主党派、工商联、无党派人士、人民团体和社会各界人士，向中省市直各单位，向驻军指战员、武警部队官兵和公安干警，表示衷心的感谢并致以崇高的敬意！

　　过去的一年，是我市各种矛盾凸显、各类问题叠加的一年，任务之重、挑战之多、压力之大，特别是经济下滑之严重前所未有。受全国煤炭产能过剩、工业领域通缩压力加大、房地产投资持续低位运行、企业融资难融资贵等因素的影响，主要经济指标大幅下降；城市转型步伐不快，经济发展中结构性、体制性和市场化程度等深层次矛盾仍比较突出；经济发展方式亟待转变，科技创新能力弱、企业竞争力不强的问题仍然存在；大项目储备少、带动作用不强，资源精深加工不够，若干产业领域的增长潜力还没有充分释放出来；财政严重短收、刚性支出增加，城市建设管理、公共服务和民生改善与人民群众的期盼还有一定的差距；安全生产和煤矿整顿关闭的压力还很大；个别领导干部精神不振，执行力不强，工作重部署、轻落实，虎头蛇尾、有始无终，推动解困发展的能力不强，突破重点难点的办法不多；经济发展环境还有待进一步优化；个别单位部门服务意识不强、办事效率不高、工作作风不实，行政效能和作风建设有待进一步加强。对于上述问题，我们必须敢于正视和面对，并将以更加有力的措施，凝聚和调动各方力量，变压力为动力、化挑战为机遇，推动问题解决，迅速扭转经济社会发展的被动局面！

　　二、2015年工作安排

　　2015年，是“十二五”规划的收官之年，也是我市解困发展、转型升级的关键之年，做好今年的各项工作意义重大。

　　今年政府工作的总体要求是：认真贯彻落实党中央、国务院和省委、省政府的一系列重大决策部署，按照市委十一届七次全会的要求，积极主动地认识和把握新常态，以稳中求进为总基调，以改革创新为主动力，以法治建设为重要保障，坚持围绕“四抓”布局，深入落实“五大规划”，科学实施“五大战略”，全面推进“十增行动”，着力加快产业项目建设，着力推进经济稳定增长，着力保障改善民生工作，着力抓好城市功能完善，着力维护社会稳定和谐，奋力开创鹤岗各项事业发展的新局面。

　　主要预期目标是：地区生产总值增长5%左右，规上工业增加值增长9%，固定资产投资增长11%，公共财政收入增长5%以上，社会消费品零售总额增长9%，进出口总额增长10%，城镇居民人均可支配收入、农村居民人均可支配收入增长7%和10%。

　　全面做好今年政府工作，要坚持和把握好以下原则：

　　——牢牢把握总基调。坚持稳中求进总基调，是做好今后一个时期全国、全省经济工作的重要指导方针。就鹤岗而言，加快发展仍是解决一切矛盾和问题的关键所在。快发展、大发展、好发展是我市当务之急，也是全部工作的重中之重。我们必须克服困难保增长，全力以赴抓增长，千方百计促增长，确保主要经济指标实现由负转正，努力开创稳中求进、进中向好的新局面。

　　——主动适应新常态。新常态下，“消费需求”、“投资需求”、“出口和国际收支”、“生产能力和产业组织方式”、“生产要素相对优势”、“市场竞争特点”、“资源环境约束”、“经济风险积累和化解”、“资源配置模式和宏观调控方式”等九大方面发生趋势性变化。正确认识新常态、适应新常态、引领新常态，是做好经济工作的基本要求。我们必须在理念和思维上适应新常态，在领导方式和工作方法上符合新常态，只有这样才能够抢占先机、主动作为、乘势而上，推动经济平稳健康发展。

　　——持之以恒转方式。在经济发展新常态下，生产要素的相对优势，从依靠劳动力低成本向依靠人力资本质量和技术进步转变；市场竞争的特点，从以数量扩张和价格竞争为主向以质量型、差异化竞争为主转变；资源环境约束，从能源资源和生态空间相对宽松向环境承载力达到或接近上限转变。面对这些新的变化，我们必须坚持以提高经济发展质量和效益为中心，把转方式调结构放到更加重要位置，更加注重创新驱动，更加注重环境保护，更加注重资源节约和合理利用，尽快实现经济发展方式的转变。

　　——坚定不移抓改革。深化改革是解困发展、转型升级的强大动力。要处理好政府和市场的关系,使市场在资源配置中起决定性作用和更好发挥政府作用，加大重点领域和关键环节改革力度，坚决破除体制机制弊端，激发全社会的发展动力和创造活力。

　　——用足用好总抓手。“五大规划”和“国务院近期支持东北振兴意见”，涵盖了农业、工业、现代服务业、对外开放、社会民生和生态建设等各个领域，是推进鹤岗解困发展、转型升级的“总抓手”。我们必须用足用好这个“总抓手”，抓住机遇、超前谋划、狠抓落实，为我市的解困发展、转型升级提供有力支撑。

　　——坚持不懈增后劲。打基础、增后劲，事关长远发展。要以“功成不必在我”的胸襟，多做承上启下、继往开来的大事，多做攒后劲、见长效的实事，多做群众看得见、摸得着的好事，特别是在改革、发展、民生等重点工作上，要正确把握好尽力而为、量力而行的关系,既要尽最大努力，做到尽力而为,不留遗憾，又要不超越可能，做到量力而行，不留矛盾和问题。让我们的工作经得起实践、人民和历史的检验。

　　依据上述总体要求和原则，今年要重点做好7项工作。

　　（一）以产业项目建设为牵动，打造转型发展新引擎。产业项目是拉动城市转型发展的重要引擎，要以培育发展“十大产业”为重点，继续开展“三引进”和“四比竞赛”活动，举全市之力，强力实施“资源型城市产业转型攻坚三年行动计划”。

　　围绕引入发展要素上项目。通过引进技术、资金、人才等要素资源，借助外力激发内生动力，是我们推进解困发展、转型升级的重要举措。要适应招商引资新常态，按照市场经济规律，依靠项目市场机遇、效益预期、要素保障和高水平服务，把优势资源同大资金、高技术有机结合，推进资源效益最大化。要抓谋划。按照产业体系、产业链，抓项目谋划；按照线索、意向、签约，抓项目生成。重点是盯牢跨国公司、央企、大的民营集团等有实力的大企业、大财团的投资意向，力争再生成一批新的项目。要求实效。既要全面掌握我市资源状况、产业基础、招商政策等内情，也要充分了解洽谈项目发展走势、市场情况、投资条件等外情，依靠有充分准备、有专业深度的政企对接活动和社会动员机制产生合作线索，切实提高项目引进成功率。要争主动。充分发挥各县区、各部门和企业的主体作用，坚定不移实施产业链式招商，盯紧“京津冀”、“长江经济带”、“珠三角”等发达地区产业转移方向，开展定向招商、定点招商和专题招商活动，切实做到承接、引进一个项目，建立一个产业龙头，拉动一个产业发展。

　　围绕现有存量扩张上项目。现有经济存量对生成新的产业项目具有重要的基础作用。依托煤炭、石墨、绿色食品、新型建材等产业已有和能够生成上游工业产品、基础工业产品的产业项目，延伸产业链条，优化产业布局，扩大产业规模。在煤电化工产业上，继续推进煤焦化及副产品加工利用、煤制化肥、煤基多联产、煤制气、煤电等产业链，重点抓好中海油煤制化肥、征楠煤化工、鹤翔新能源、中石化煤基多联产等重大项目。在石墨深加工产业上，继续推进石墨电池负极材料、密封材料、阻燃材料、锂离子蓄电池、胶体石墨等产业链，重点抓好中铁石墨等重大项目。在绿色食品产业上，继续推进稻米、玉米、大豆深加工和畜牧养殖、林产品加工等产业链，重点抓好万源粮油等重大项目。

　　围绕引进高新技术上项目。要大力推进科技成果产业化，培育、引进技术含量高、对资源环境等要素依赖性不强的科技型企业，主动对接大专院校和科研院所，选择成长性好、发展潜力大的新技术、新成果、新专利，迅速转化成为科技含量和附加值高、有竞争力、有市场潜力的产业项目。重点推进锦城科技粉煤灰综合利用、明珠科技米蛋白、秀泉科技氧化法制浆、鹏程科技吸音板扩产等高科技项目建设，加快推进与中植集团、教育部、南开大学合作成立的南开国家新材料研究院项目的落实。

　　围绕挖掘资源优势上项目。推进矿产资源“探、采、选、冶、研、加、用”一体化、全产业链式发展，对占而不探、探而不采、采而不加、加而不深的实施资源深度整合，推动矿产资源高效利用。加快推进中铁资源集团与深圳贝特瑞、检顺公司与杉杉科技石墨合作项目，与中植集团、中国有色集团等洽谈合作的煤化工、石墨、粮油深加工项目要有新突破。充分利用丰富的农业、林下资源和寒地黑土、绿色有机的特色优势，大力发展绿色食品产业，推进资源型城市向绿色食品强市转变。积极推进华润五丰、海宇米业公司稻谷深加工及方便米饭项目。

　　（二）以农民持续增收为核心，实现三农工作新突破。要以增加农民收入为核心，进一步提高农业综合生产能力，加快农业现代化进程，让农业强起来，让农村美起来，让农民富起来。

　　着力推进基础设施建设，提高农业综合生产能力。加强水利化建设，围绕全国水生态文明城市建设试点，投资3.2亿元，重点实施防灾减灾、农村水利、水环境修复等13项水利工程。加强农机化建设，争取农机购置补贴资金900万元，新组建现代农机合作社5个，培养省级规范社3个，建设千亩农机科技示范园区21个，田间综合机械化程度达到98%以上。加强科技化建设，全市标准化生产面积要达到300万亩，农业先进适用技术推广面积达到900万亩（次），良种覆盖率达到98%以上。新建智能水稻浸种催芽车间4处、育秧大棚1544栋。切实加强农业气象服务与农村气象灾害防御体系建设。支持宝泉岭农垦管理局的改革发展，发挥其示范带动作用，提高域内粮食产能。

　　着力推进经营方式创新，构建新型农业经营体系。要培育多元农业经营主体，加快农地“三权分离”，推进农村土地承包经营权确权试点工作，健全公开规范的土地流转市场，鼓励土地承包经营权在公开市场上向专业大户、家庭农场和农民合作社流转。

　　着力推进富民产业发展，拓宽农民持续增收渠道。巩固种植结构调整成果，农作物总播种面积要稳定在333万亩，其中高产高效作物面积310万亩。大力发展绿色种植，面积达到200万亩，“三品”标识总量达到300个。大力发展畜牧水产业，创建水产、畜禽标准化养殖场9个，网箱、网栏养殖面积达到4.5万平方米，名特优水产品养殖面积达到4.1万亩。大力发展花卉、瓜果、马铃薯、饲料饲草等特色种植。发挥鹤北林业局龙头带动作用，大力发展林木加工业和食用菌、北药、红松坚果、山野菜等林下经济，启动省级林业产业科技示范园区建设。推进菜篮子工程建设，蔬菜种植面积达到4万亩，新建高标准温室大棚200栋。搭建劳转服务平台，促进农村劳动力转移和劳务收入逐步提高。

　　（三）以促进转型升级为方向，培育工业经济新优势。工业的“脊梁”不硬，财政的“身板”就挺不起来。坚持活化存量与引进增量并举，扩张总量与提升质量并重，切实把工业转型升级作为中心任务，努力增强工业经济综合实力。

　　抓运行活存量。要抓政策落实。在不折不扣地用足用好国务院35条、省政府65条和市委市政府50条等支持政策的同时，跟踪研判国内外经济走势，及时向企业传导国家和省的各类支持政策和措施，让稳增长的政策效应最大化。要抓运行调控。完善现场办公、集中会商工作机制，严格落实各级领导、各部门包企责任制，全面掌握并及时解决企业运行中遇到的各类难题，通过抓好现有企业运行，稳一块；通过加强对已建成和即将建成项目的服务，增一块；通过支持重点企业加快改造升级，强一块；通过加大对停产半停产企业包保力度，活一块。要抓统筹推进。牢固树立“一盘棋”思想，举全市之力，大力支持龙煤鹤岗矿业公司发展。无论是对中省直企业还是对地方企业，无论是对骨干重点企业还是对小微企业，都要一视同仁，尽最大努力支持其发展。

　　抓调整扩增量。坚定不移实施工业结构的战略性调整，以“三大主导产业”为牵动，培育新兴产业和发展替代产业，大上优势产业项目、新能源和新材料项目、循环经济和低碳经济项目。重点实施68个工业项目，其中，亿元以上项目24个。确保竣工投产23个，使新增工业规模占现有工业规模的三分之一以上。

　　抓创新提质量。扶持骨干企业实施技术创新、技术改造和结构升级，推进与院校、科研机构、高科技企业嫁接，积极开发新产品，实现产品升级换代。加强企业自主创新能力建设，推动各类创新要素向企业聚集，引导企业加大研发设计投入，开发拥有自主知识产权的新技术、新产品，增强企业核心竞争力。以全省实施“千户科技型企业三年行动计划”为契机，全力打造科技型中小企业集群，三年培育科技型企业100户，带动产业转型升级。

　　（四）以市场体系建设为引领，强化第三产业新支撑。面向国内国际两大市场，不断提升商贸流通、金融、对外经贸和旅游、文化产业的规模和水平，增强第三产业对整体经济的拉动作用。

　　打造专业市场，做强商贸流通业。扶持比优特时代广场、宏益家电、红星美凯龙等企业发展壮大，积极引入域外大型商业集团。加快南翔、陆港、加兴等物流项目建设。推进大型农副产品市场建设，引导大型连锁超市、流通企业与农副产品专业合作组织建立直接采购基地，促进产销衔接。支持企业借助产品品牌和渠道品牌，通过旗舰店、电商网络渠道建立营销网络。

　　打造特色精品，做优旅游业和文化产业。加快发展旅游产业，推进旅游客运集散中心、旅游会展中心等公共服务设施建设以及金顶山、龙湖和鹤立湖等景区升级。组建鹤岗·黑龙江三峡旅游集团总公司，对太平沟、名山岛、月牙湖3个国家4A级旅游景区进行市场化运营，培育鹤岗·黑龙江三峡5A级旅游景区。加快发展文化产业，推进漫狐动漫网络游戏及鹤岗“6公里”生活服务圈项目；抓好名山和金羽丝路文化创意产业园建设，促进文化产业与科技、信息、旅游等产业的融合发展。

　　打造融资平台，做活现代金融业。继续实施“引金入鹤”工程，鼓励金融机构创新业务品种和服务模式，打造多层次现代金融服务平台。加快组建鹤岗市农商银行，积极筹建融资租赁公司、投融资公司和融资担保公司。进一步创新投融资体制，采取PPP投融资模式，吸引更多的社会资本参与基础设施、公用事业等领域投资建设，解决发展资金短缺问题。推进万源粮油、经纬木糖、泰丰米业、华升救援等企业在“新三板”上市取得实质性进展。

　　打造开放前沿，做大对外经贸产业。按照“出口抓加工、进口抓落地”的思路，加快对外经贸主体企业培育，巩固提升农药、特色农产品等传统出口产品档次，培育壮大石墨等优势产品出口规模，开发欧美、拉美、东南亚、中东和非洲等国家和地区的市场。抓住我省推进东部陆海丝绸之路经济带建设的有利契机，积极应对俄远东对外经贸政策的变化，适时启动中俄经贸合作区建设，推进赴俄锰铁矿开发项目竣工投产，扩大赴俄开发种植面积。引进域外大型电商企业投资跨境电子商务，全力打造跨境电子商务试点城市和电子商务示范基地。

　　（五）以增强承载能力为目标，构建城市发展新格局。坚持以科学编制规划为引领，以完善城市功能为重点，以城市经营和加强精细化管理为突破口，进一步增强城市对经济社会发展和人民生产生活的综合承载能力。

　　实施规划修编工程。要牢固树立“规划先行”的理念，以编制“十三五”规划为契机，全面完成城市总体规划、土地利用总体规划和水、路、电、气等基础设施规划修改工作，高质量编制城市工矿区搬迁及煤矿棚改房、沉陷区搬迁安置房、城市棚户区改造建设等规划。进一步完善产业园区规划，加快园区提档升级。

　　实施路网改造工程。要全面推进公路、铁路、空路、水路“四路同建”，使鹤岗尽快融入到全国互联互通之中，解决区位劣势、“神经末梢”的问题。积极推进绥名公路、萝额公路鹤岗过境段、鹤大公路出口段等项目，加快规划鹤伊公路提档升级；大力推动鹤佳铁路扩能改造和园区铁路建设；全力争取鹤岗机场建设项目纳入国家机场建设规划；加快名山港码头扩建和海关监管库建设步伐，打通鹤岗入海口，实现江海联运。进一步推进城区道路升级改造，投资1.5亿元，新建改造10条主次干道，硬化维修53条街巷道路，打通一批卡脖路、断头路。

　　实施“四供”保障工程。在供水上，启动市政供水系统升级改造工程，新建农村饮水安全工程43处，解决2.48万人的饮水安全问题。完成关门嘴子水库项目建议书批复，全面启动可研工作。在供电上，深入实施农网改造和110千伏南河线等工程，重点推进鹤岗500千伏输变电工程。在供热上，加快热源建设，推进热力公司四期工程、2x300MW热电联产项目和老旧管网改造。在供气上，完成部分燃气老旧管网改造，新增用户5000户。

　　实施综合整治工程。全力抓好市容市貌管理，严格落实“门前三包”，实施“八乱”现象整治行动，实现城市主次干道“全天候”保洁和“无缝隙”管理。全面提高城市绿化水平，植树8.7万株，重点推进麓林路、东山路等道路、公园、广场的绿化美化。做好街巷道路亮化，确保亮灯率达到98%以上。深入开展“弃管楼”整治，严把物业企业资质审批关，严格执行新建住宅项目履约保证金、房屋共用设施质量保证金制度。要加大土地、建筑、房地产市场整治力度，依法坚决打击建设用地、建筑质量、房屋征收拆迁和房屋销售等方面的违法违规行为。

　　实施环境保护工程。继续推进主要污染物减排工程，化学需氧量、氨氮、二氧化硫和氮氧化物排放量分别比2010年削减14%、13.5%、2%和4.5%。落实大气污染防治行动计划，继续淘汰燃煤小锅炉、黄标车及老旧车辆。开展环境空气PM2.5自动监测，实时发布监测信息。加快推进生活垃圾处理工程，做好垃圾收运系统建设。实施市区污水管网工程，提升污水并网率，城市水环境功能区水质达标率达到100%。推进生态文明建设示范村镇创建，加快农村环境综合整治，力争3个乡镇晋升为国家级生态乡镇。

　　（六）以保障改善民生为根本，开创各项事业新局面。坚持可用财力向民生投入、工作力量向民生倾斜，统筹抓好10个方面工作，把老百姓期盼的事情办实办好。（1）统筹抓好就业创业。就业是民生之本，要落实更加积极的就业创业政策，加快人力资源市场建设，加大小额担保贷款、创业培训扶持力度，建立政府激励创业、社会支持创业、劳动者勇于创业的长效机制，掀起全民创业新高潮。实现新增就业2.3万人，下岗再就业1.5万人，城镇登记失业率控制在4%以内，高校毕业生就业率达到85%以上。（2）统筹抓好城乡人居条件改善。这项工作，既是群众所盼，也是政府面临的一项长期性、艰巨性、紧迫性的任务，不管多难也要办好。要加快保障性安居工程建设，城市棚户区新建7628户、续建1102户，回迁安置1102户；煤矿棚户区新建10160户、结转续建1493户，回迁安置4000户；结转续建公共租赁住房3900户；加快解决水淹区和一氧渗出区居民的住房问题；加快推进公职人员小区建设，确保年底竣工。（3）统筹抓好城乡社会保障体系建设。社会保障是民生之基、安全之网，要坚持全覆盖、保基本、多层次、可持续方针，继续扩大“五险”覆盖面，适时提高城乡低保补助标准，达到人均每月470元。要积极筹措资金，做好各类困难群众救助工作，尤其是重大传统节日期间，将筹资1亿元用于社会救助，切实保障困难群众基本生活。（4）统筹抓好社会养老服务。要积极应对人口老龄化的趋势，发展养老服务业，加强机构养老、社区养老和居家养老，办好公办保障性、民办公益性的养老服务机构，每千名老人拥有养老床位达到32张，居家养老服务达到全覆盖，实现老有所养。（5）统筹抓好社区建设。要全力推进城乡“双百”社区建设，创建“双百”社区4个，努力为群众提供最贴心、最周到的公共服务，让社区成为群众安居乐业的和谐家园、幸福家园。（6）统筹抓好教育事业。要优化配置教育资源，不断加强基础教育、高等教育、职业教育和特殊教育、民办教育，提高教育质量，进一步推进各级各类教育均衡发展。推进2所标准化学校和4所学校信息化建设，投入1000万元建设职教中心综合楼，创建国家级空飞空乘培训基地。加大教师队伍建设力度，着力解决教师队伍结构性缺编问题。完善助学体系，资助家庭贫困学生9000人，让每一个孩子都能上得起学、上得好学。（7）统筹抓好卫生计生事业。深化医药卫生体制改革，引进社会资本，推进健康服务业发展。优化配置医疗卫生资源，组建医疗联合体。大力提升卫生综合服务能力，确保市妇幼保健院、鹤岗卫校农村医学综合楼投入使用。深入开展“健康龙江行动”和“医疗服务质量提升年”活动，为群众提供方便、快捷、优质、高效的医疗卫生服务。全面落实卫生计生各项惠民政策措施，兑现农村及城镇无业人员独生子女奖励费。（8）统筹抓好文化事业。要继续推进文化惠民工程，着力完善城区公共文化基础设施，全面完成市县两级博物馆、图书馆、文化馆达标建设，推进社区文化活动中心、文化活动室建设，继续推动公共文化设施免费开放，开通地面数字电视节目，丰富活跃群众文化生活。（9）统筹抓好体育事业。不断加强群众身边体育设施建设，广泛开展全民健身运动，进一步提高竞技体育水平，办好全市第十届运动会暨第二届全民健身运动会，积极备战省第十四届运动会和全国第十三届冬运会，启动公职人员小区康体中心建设。（10）统筹抓好科技人才事业。要大力推进科技兴市战略，加强科技综合服务平台建设，为全市科技型企业和人才创新创业、技术引进、成果转化、科技合作提供全方位服务。全力促进企业研发机构建设，引导、鼓励企业与高校院所建立研发机构，促进产学研深度融合。要大力实施人才强市战略，认真落实引进、培养人才的倾斜政策。注重引进科技研发、经营管理、资本运作、旅游开发、项目策划等方面人才，招录一批优秀大学毕业生、研究生，促进人才结构年轻化、素质综合化。完善“鹤岗籍在外人才库”，建立柔性引进人才机制，有效缓解人才紧缺困境。完善本土人才培养机制，激发各类优秀人才的积极性。注重培养企业领军人才，加快政府智库建设，为我市的转型发展提供人才和智力支持。

　　（七）以平安鹤岗建设为重点，营造和谐稳定新环境。推进平安鹤岗建设，关乎经济社会发展，关乎社会和谐稳定，关乎人民群众切身利益，必须持之以恒、长抓不懈。

　　加强安全生产工作。要突出抓好煤矿安全，加快地方煤矿整顿关闭工作，确保复产煤矿全部达标生产。严格落实党政同责，强化“四个责任”，坚持24小时“旁站式”监管和“五化一加强”、“六防”等各项制度，确保煤矿安全生产。切实抓好消防安全，坚决整治东北亚商城等人群密集场所安全隐患，落实各项防范保障措施，坚决避免火灾事故发生。全力推进食品药品安全“社会共治”，健全并严格执行产品质量安全标准。建立覆盖城乡的食品药品、农产品质量监测体系。全力抓好特种设备、交通运输、非煤矿山、危险化学品、建筑行业等重点领域的安全。

　　加强信访稳定工作。要落实信访包案责任制，完善三级信访终结制度，建立健全信访信息反馈报告制度。进一步畅通群众诉求渠道,探索创新依法处访的新途径,实施信访案件分类办理和倒查机制,妥善解决好群众关心的热点和难点问题。

　　加强社会治安综治工作。要依法严厉打击各种刑事犯罪和经济犯罪，继续开展严打整治专项行动，创新警务模式，推进警力下沉，不断提升人民群众的安全感和满意度。加强政府应急指挥平台建设，完善各类事件应急预案，进一步提高应急处置能力。

　　加强诚信体系建设工作。要加快启动社会诚信体系建设规划，全面推动政务诚信、商务诚信、社会诚信和司法公信建设，建立健全覆盖全社会的征信系统，完善政府、企业和个人信用体系，建立黑名单制度和信用奖惩制度，提高失信成本。

　　各位代表，做好政府工作，必须以改革创新精神加强自身建设。要巩固党的群众路线教育实践活动成果，坚决落实各项整改措施，时刻牢记最好的工作是只争朝夕，最好的落实是一抓到底，最好的行动是说到做到，努力建设人民满意政府。一要振奋精神，坚定解困发展信心。2015年仍将是我市经济社会发展较为艰难的一年，越是面对压力和挑战，就越需要政府一班人坚定信心、迎难而上，团结一致、共克时艰，心往一处想、力往一处聚、劲往一处使，遇到急难险重不绕弯，面对阻力矛盾不回避，为推动我市经济解困、转型发展汇聚强大动力。二要求真务实，切实做到履职尽责。牢固树立正确的政绩观，坚持做到重实际、说实话、出实招、求实效，坚决不搞脱离实际的“数字”、沽名钓誉的“形象”、华而不实的“作秀”和劳民伤财的“政绩”。要脚踏实地干工作、尽心尽力履职责，一心一意谋发展、真心实意惠民生，以广大干部的具体行动和实际工作，落实本届政府的使命担当。三要依法行政，自觉接受各界监督。主动适应依法治国新要求，自觉运用法治思维、法治理念、法治程序、法治方式来加快建设法治政府。要依法规范政府职权，树立强烈的法治意识、制度规则意识、信用意识、权责对等意识，做到“法定职责必须为、法无授权不可为”，不断提高政府的公信力和执行力。要健全依法决策机制，把公众参与、专家论证、风险评估、合法性审查、集体讨论决定，确定为重大行政决策法定程序，建立行政机关内部重大决策合法性审查机制、重大决策终身责任追究制度及责任倒查机制，依法规范重大资金使用制度、重大问题决策机制。要坚持严格规范公正文明执法，积极推进行政执法体制改革，在条件成熟的领域实行跨部门综合执法，进一步规范征地补偿、征收拆迁行为，加大食品药品、安全生产等重点领域的执法力度，维护群众合法权益。要自觉接受对政府权力的制约和监督，主动接受人大监督、民主监督、行政监督、审计监督、社会监督和舆论监督，大力推进政务公开，发挥好企业投诉中心作用，坚决查处损害经济发展的人和事，进一步改善发展环境。四要强化执行，狠抓工作任务落实。牢固树立刚性化、绩效化、精细化的执行理念，强化执行的过程控制，建立健全抓落实的目标体系、责任体系和督查体系，无论是制约长远发展的重大问题，还是群众反映强烈的难点、热点问题，都要编制路线图、确定时间表、落实责任人，以目标倒逼进度、以督查倒逼落实，确保重大决策、重大项目、重点工作不折不扣落实到位。要继续加强基础性工作，坚持工作专业化标准，研究问题、推动工作都要有具体路径。对各项工作部署都要狠抓落实，严肃追究失职渎职责任，做到奖罚分明。五要干净干事，加强廉洁政府建设。要认真贯彻落实党风廉政建设责任制，严格履行“一岗双责”，自觉遵守廉洁自律的有关规定，加强对公共资金、国有资产、国有资源、宏观政策措施落实、领导干部履行经济责任情况的审计监督，严肃查处违法违纪案件，让权力在阳光下运行。牢固树立过“紧日子”思想，认真执行《厉行节约反对浪费条例》、《国内公务接待管理规定》等法规制度，严格控制“三公”经费，集中财力保民生、促发展。

　　各位代表，鹤岗正处在解困发展、转型升级的关键时期。我们任务艰巨、责任重大、使命光荣，让我们在省委、省政府和市委的坚强领导下，紧紧团结和依靠全市各级党政组织和广大干部群众，以只争朝夕的精神、奋发昂扬的姿态、坚韧不拔的毅力、真抓实干的作风，迎接挑战，主动作为，改革创新，攻坚克难，奋力开创鹤岗各项事业发展的新局面！
